# A case report of IgG4-related hepatic inflammatory pseudotumor in a 3-year old boy

**DOI:** 10.3389/fimmu.2024.1376276

**Published:** 2024-04-30

**Authors:** Qian Wan, Zhongjin Xu, Xiaohui Liu, Zhuqiang Wu, Qingmei Zhong, Chongjun Wu

**Affiliations:** ^1^ Department of Hematology, Jiangxi Provincial Children’s Hospital, Nanchang, China; ^2^ Department of Rheumatology and Immunology, Jiangxi Provincial Children’s Hospital, Nanchang, China; ^3^ Nuclear Magnetic Resonance Room, Jiangxi Provincial Children’s Hospital, Nanchang, China; ^4^ Department of Pathology, The Ninth Hospital of Nanchang, Nanchang, China

**Keywords:** hepatic inflammatory pseudotumor, IgG4-related disease, steroid, child, liver biopsy

## Abstract

**Background:**

Hepatic Inflammatory Pseudotumor (IPT) is an infrequent condition often masquerading as a malignant tumor, resulting in misdiagnosis and unnecessary surgical resection. The emerging concept of IgG4-related diseases (IgG4-RD) has gained widespread recognition, encompassing entities like IgG4-related hepatic IPT. Clinically and radiologically, corticosteroids and immunosuppressive therapies have proven effective in managing this condition.

**Case Presentation:**

A 3-year-old Chinese boy presented to the clinic with an 11-month history of anemia, fever of unknown origin, and a tender hepatic mass. Blood examinations revealed chronic anemia (Hb: 6.4 g/L, MCV: 68.6 fl, MCH: 19.5 pg, reticulocytes: 1.7%) accompanied by an inflammatory reaction and an elevated serum IgG4 level (1542.2 mg/L). Abdominal contrast-enhanced computed tomography unveiled a 7.6 cm low-density mass in the right lateral lobe, while magnetic resonance imaging demonstrated slight hypointensity on T1-weighted images and slight hyperintensity on T2-weighted images, prompting suspicion of hepatic malignancy. A subsequent liver biopsy revealed a mass characterized by fibrous stroma and dense lymphoplasmacytic infiltration. Immunohistochemical analysis confirmed the presence of IgG4-positive plasma cells, leading to the diagnosis of IgG4-related hepatic IPT. Swift resolution occurred upon initiation of corticosteroid and mycophenolate mofetil therapies.

**Conclusion:**

This study underscores the diagnostic approach to hepatic IPT, utilizing histopathology, immunostaining, imaging, serology, organ involvement, and therapeutic response. Early histological examination plays a pivotal role in clinical guidance, averting misdiagnosis as a liver tumor and unnecessary surgical interventions.

## 1 Introduction

Hepatic Inflammatory Pseudotumor (IPT) stands as an infrequent benign lesion often mistaken for a malignant tumor, leading to unnecessary surgical resections. Characterized histologically by the proliferation of fibroblasts or myofibroblasts and the presence of inflammatory cells, primarily polyclonal lymphocytes and plasma cells, Hepatic IPT accounts for approximately 8% of extrapulmonary IPT cases (1). With the emergence of the concept of immunoglobulin G4 (IgG4)-related hepatic IPT (2), interest in studying this rare condition has surged. Notably, only a handful of cases, particularly in children, have been reported, limiting our understanding of this disease (3). We present a unique case of IgG4-related hepatic IPT in a 3-year-old boy who sought medical attention with an 11-month history of anemia, a fever of unknown origin, and a tender hepatic mass. Previous cases, such as those reported by Al-Hussaini et al. (4)and Shibata et al. (5), underscore the challenge in accurately diagnosing hepatic IPT, often leading to recommendations for surgical interventions due to a high suspicion of malignancy. In this case, immunohistochemical analysis revealed focal positivity for IgG4, prompting consideration of IgG4-related hepatic IPT as part of the IgG4-related diseases (IgG4-RD) hepatobiliary disease spectrum (3). Given the rarity of this condition and the potential for misdiagnosis, we advocate for increased awareness and consideration of IgG4-related hepatic IPT in pediatric patients presenting with similar clinical features.

In order to avert diagnostic oversight and consequent therapeutic delay in this condition, we present a rare case of IgG4-related hepatic IPT in a 3-year-old male patient. The child sought medical attention with an 11-month history marked by anemia, a fever of unknown origin, and a tender hepatic mass. Significantly, successful resolution of the disease was achieved through a course of corticosteroid and mycophenolate mofetil therapy.

## 2 Case presentation

A 3-year-old male presented at the clinic with an 11-month history characterized by anemia, a fever of unknown origin, and a tender hepatic mass. Initial routine blood examination 11 months ago revealed anemia (hemoglobin 82 g/L), while abdominal Doppler ultrasound and chest X-ray results displayed no abnormalities. Notably, further investigation into the cause of anemia was not pursued at that time. Subsequently, iron supplementation was initiated, but after one month of treatment, no improvement in anemia was observed. Upon admission to our hospital, the patient exhibited hemoglobin levels of 66 g/L, prompting a thorough examination for diagnosis and treatment. Additionally, the past 3-month medical history included significant abdominal distention, interpreted as indigestion and managed with probiotics by the parents. There was no consanguinity, chronic illness, or malignancies in the family history. The physical examination was notable for tender hepatomegaly, palpable 3 cm below the costal margin. The physical examination revealed notable pale complexion and tender hepatomegaly, palpable 3 cm below the costal margin. Several enlarged lymph nodes were palpated on both sides of the neck, armpits, and groin, with the largest being about 2×1cm, no rupture and acceptable mobility. Rash or subcutaneous mass were not found on the entire body skin. Swollen mass was not found in the orbit, tonsils, or nose. Bilateral parotid and submandibular glands were not enlargement. Physical examination of the heart, spleen, pancreas, and kidneys showed no abnormalities. Major vascular murmurs or abnormal breathing sounds were not found.

Laboratory investigations unveiled chronic iron-deficiency anemia in the patient, characterized by a hemoglobin level of 6.4 g/L, MCV of 68.6 fl, MCH of 19.5 pg, reticulocyte count of 1.7%, ferritin concentration of 87.7 ng/mL (within the normal range of 50-142 ng/mL), and a serum iron level of 5.8 μmol/L (normal range: 13-32 μmol/L). Folic acid, vitamin B12, and thalassemia gene levels were found to be within normal limits. Bone marrow cytology revealed proliferative anemia, with 1% intracellular iron and + extracellular iron.

Other laboratory tests showed a normal white blood cell count, renal, hepatic, coagulation profiles, a normal eosinophils number of 0.05×10^9^/L, a normal eosinophil percentage of 0.4%, a high erythrocyte sedimentation rate (ESR) of 76 mm/h, a high immunoglobulin G (IgG) concentration of 60.74 g/L, a normal IgE concentration, a normal complement 3 concentration, a low complement 4 concentration of 0.11g/L, a high C-reactive protein (CRP) level of 236.5 mg/L and a high fungal D-glucan level of 974.6 pg/mL. Serological tests for hepatitis-B, syphilis, Epstein-Barr virus (EBV), and human immunodeficiency virus were negative. Tumor markers such as alpha-fetoprotein (AFP) and carcinoembryonic antigen (CEA) were negative.

Abdominal Doppler ultrasound revealed a heterogeneous lesion measuring 9.1 cm × 6.6 cm × 6.9 cm in the right hepatic lobe, displaying a nonspecific appearance.

**Figure 1:**
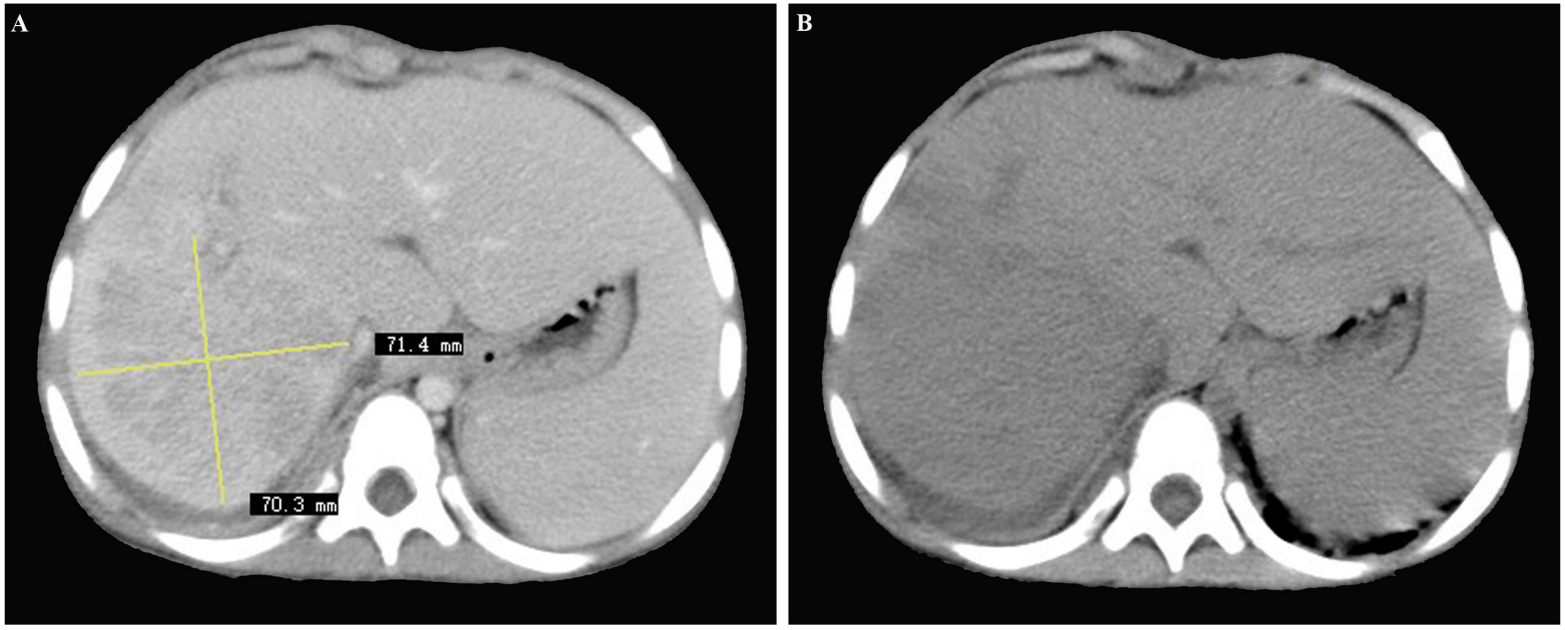
**(A, B)** Contrast-enhanced computed tomography scan of the abdomen revealing a poorly-defined, mixed-density lesion (7.0 cm × 7.1 cm) with internal enhancement involving the right hepatic lobe, initially considered as hepatic malignancy.

Contrast-enhanced computed tomography (CT) of the abdomen revealed a poorly-defined, mixed-density lesion (7.0 cm × 7.1 cm) with internal enhancement involving the right hepatic lobe, suggestive of hepatic malignancy (**Figure 1**). In the delayed venous phase, both the portal vein and inferior vena cava were patent.

**Figure 2:**
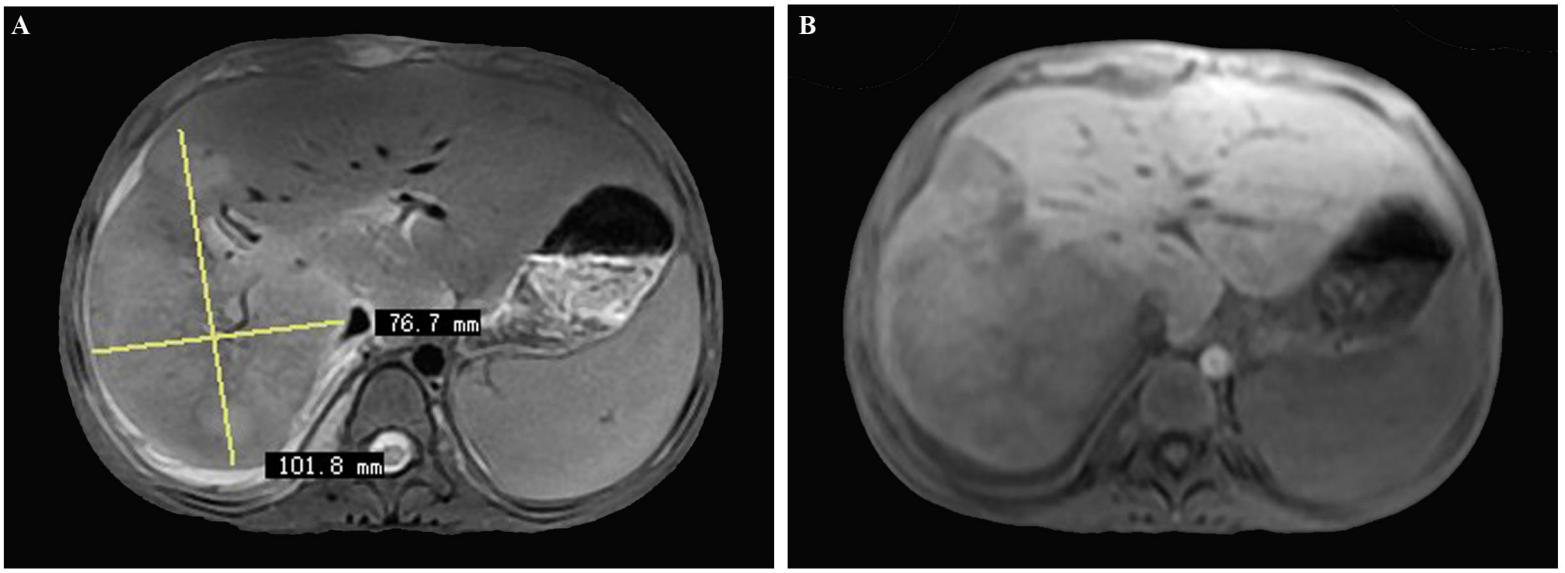
**(A, B)** Magnetic resonance imaging (MRI) of the abdomen displaying a 10 cm × 7.6 cm, contrast-enhancing, poorly defined, large-sized lesion involving the right hepatic lobe, also initially considered as hepatic malignancy.

Magnetic resonance imaging (MRI) of the abdomen displayed a 10 cm × 7.6 cm, contrast-enhancing, poorly defined, large-size lesion affecting the right hepatic lobe, also indicative of hepatic malignancy (**Figure 2**).

Ultrasound of bilateral lacrimal gland, salivary gland, parotid gland, submandibular gland, and thyroid did not show enlarged glandular. Ultrasound of retroperitoneum, pancreas, bile duct, kidney, and spleen showed no abnormalities. Superficial lymph node ultrasound showed enlarged lymph nodes in both necks, with the largest being about 18×7mm on the left side, 19×8mm on right side, several small lymph nodes under both armpits, with the largest being about 7×3mm, several lymph nodes in bilateral inguinal region, with the largest being about 8×3mm. The lymph nodes were with clear boundaries between the cortex and medulla. CT of pulmonary suggested infection and diffuse interstitial changes in both lungs. No abnormalities were found in the MRI of the orbit, head, and pituitary gland.

**Figure 3:**
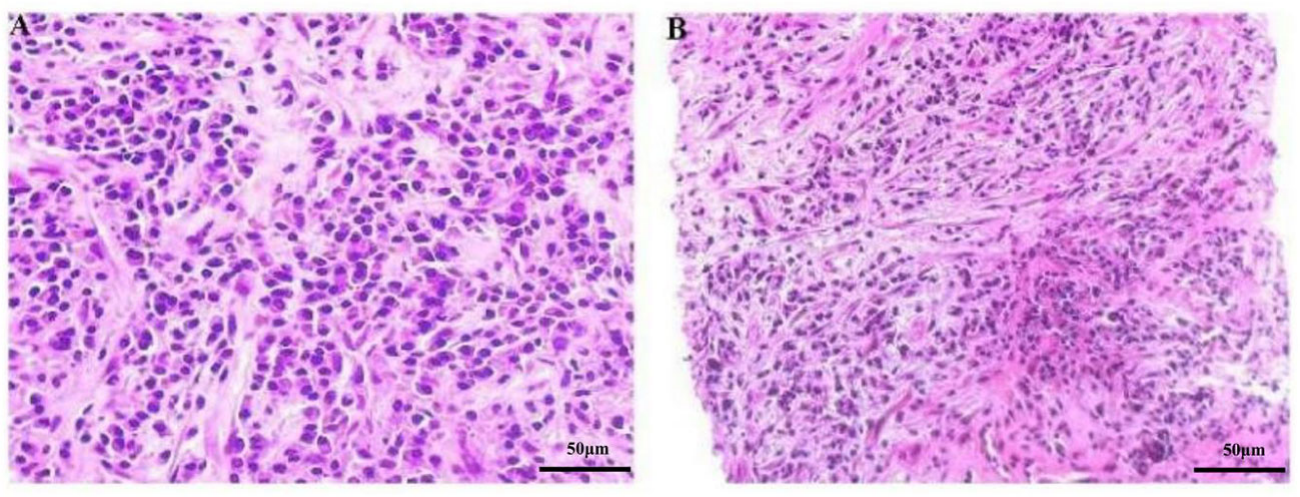
**(A, B)** Microscopic image of the focal hepatic lesion showing a mixture of inflammatory cells in a background of dense fibrous tissues (HE stain, magnification power: ×200).

**Figure 4:**
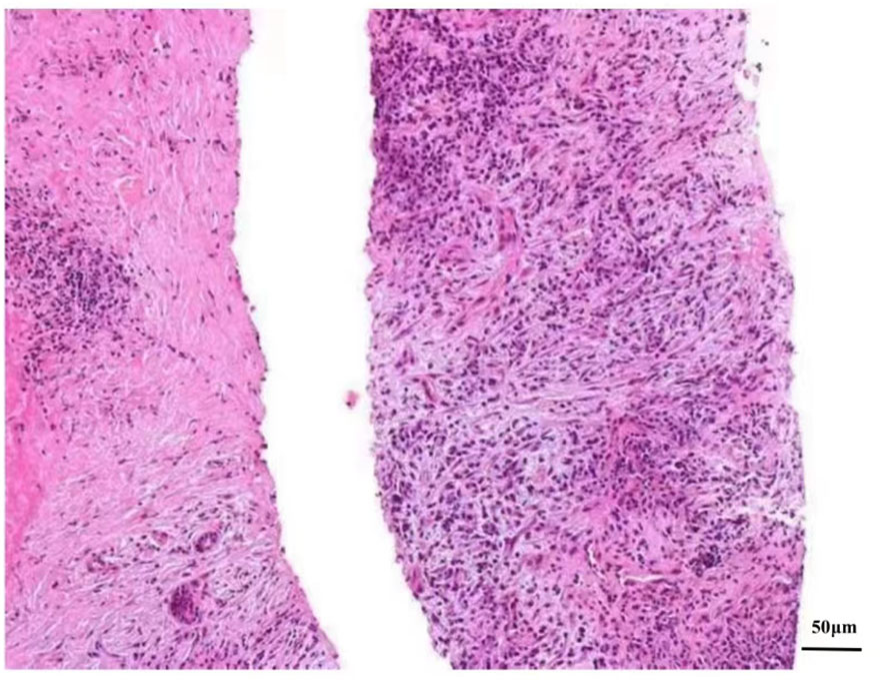
Microscopic picture of the focal hepatic lesion displaying obliterating phlebitis (He stain, magnification power, ×200).

Ultrasonography (US)-guided percutaneous needle biopsy (Pro•Mag™, 18 gauge, 10 cm, Athens, TX75751, USA) was performed on the hepatic mass. Histopathological analysis revealed dense fibrous tissue around the portal area in storiform, along with a mixture of inflammatory cells and eosinophil infiltration in the portal area (**Figure 3**). Obliterating phlebitis was observed, while lymphoid follicles were absent (**Figure 4**). The biopsy showed no granulomas or necrotic areas. Immunohistochemically, the inflammatory cells stained diffusely positive for CD38 and Mum-1 (**Supplementary Figures 1A, B**), indicative of plasma cells. Additionally, the hepatic lesion displayed focal positivity for IgG4 [> 10 plasma cells/high-power field(HPF)] (**Supplementary Figures 1C, D**), with a ratio of IgG4/IgG plasma cells exceeding 40%, meeting the criteria for classification as an IgG4-related disease. The serum IgG4 concentration was markedly elevated at 1542.2 mg/L (normal range: 10-537 mg/L).

Based on a history of recurrent respiratory tract infections, CT of pulmonary, and elevated levels of fungal D-glucan, we considered that the lung lesions of the patient in our case is fungal pneumonia. After 2 weeks of treatment with voriconazole, CT of pulmonary showed significant improvement and fungal D-glucan levels decreased to normal. Upon integration of the clinical and radiological features, serum IgG4 levels, and pathological features, a conclusive diagnosis of IgG4-related hepatic IPT(lymphoplasmacytic type) was established. Subsequently, treatment commenced with mycophenolate mofetil (0.25g/day) and prednisone (1mg/Kg/day). At the 8-month postoperative of liver biopsy follow-up, the patient remained afebrile and asymptomatic, with ultrasound evidence indicating a reduction in hepatic mass (2.9 cm × 2.3 cm × 1.8 cm) and an increase in hemoglobin levels (112g/L at 8 months).

## 3 Discussion

IgG4-RD represents a rare fibroinflammatory disorder associated with chronic, yet unidentified, antigenic stimulation. Up until 2022, a mere 22 cases of pediatric IgG4-RD have been documented, with the median age at initial symptom onset reported as 13 years (ranging from 22 months to 17 years) (6). Within the spectrum of diverse organ manifestations observed in pediatric IgG4-RD, the occurrence of IgG4-related hepatic inflammatory pseudotumor as an organ manifestation has been delineated in only one prior case report involving a ten-year-old child (7, 8). The present report contributes to the limited literature on pediatric IgG4-RD by detailing the case of a 3-year-old boy exhibiting hepatic inflammatory pseudotumor as a manifestation of IgG4-RD.

The symptoms of IgG4-RD are variable and contingent on the affected organs. However, the clinical presentation of hepatic IPT is characterized by nonspecific features, including abdominal pain, fever, weight loss, and general fatigue, which commonly lead patients to seek evaluations across various departments in pursuit of a diagnosis (9).We listed the past IgG4-related IPT involving liver in **Table 1** (5, 10–16). **Table 1** details previous cases of IgG4-related inflammatory pseudotumors involving the liver (5, 10–16). Specifically, the patient in this report exhibited leading symptoms of anemia, fever, and a tender hepatic mass within the hematology department. Laboratory examination of the anemia revealed hypochromic microcytic anemia. The reason for anemia was considered to chronic consumption of the gigantic hepatic mass of the patient. The final reason remains to be further studied. Notably, two cases in children reported by Wiegering et al. emphasized anemia as a primary symptom for hepatic IPT (17), with hypochromic microcytic anemia, a laboratory indicator of an inflammatory response, also documented in pediatric hepatic IPT cases (18). The presentation of our case contributes to raising awareness of IgG4 RD in pediatrics, particularly among hematologists.

**Table 1:** Studies reporting on IgG4-related inflammatory pseudotumor involving liver.

Reference	Age	Gender	Tumor site	Prognosis	Treatment
Itazaki Y 2021 (10) Kim F 2011 (11) Shibata M 2016 (5) Miyajima S 2017 (12) Okamura Y 2021 (13) Mulki R 2015 (14) Kanno A 2005 (15) Lee JH 2018 (16)	75y 58y 72y 50y 67y 50y 48y 67y	F M M F M M M M	Left liver Kidney and Liver Liver Liver and abdominal wall Left hepatic umbilical portion Left liver Liver Liver	IgG-R IPT IgG-R IPT IgG-R IPT IgG-R IPT IgG4-SC IgG-R IPT AIP with IP IgG4-R pseudotumor	Surgical excisions Prednisolone Corticosteroids Corticosteroids Surgical excisions Surgical excisions Corticosteroids Steroid and ursodeoxycholic acid tablet

Y, year; M, male; F, female; IgG-R IPT, IgG4-related inflammatory pseudotumor; IgG4-SC, IgG4-related sclerosing cholangitis; AIP, autoimmune pancreatitis; IP, inflammatory pseudotumor.

The initial misdiagnosis in our case stemmed from the nonspecific nature of the clinical presentation and radiological findings. Discriminating hepatic IPT from malignant tumors, particularly in children given their rare incidence, poses a significant challenge. Based on the CT and MRI results, we initially categorized the hepatic mass in this case as hepatic malignancy (19). Similarly, contrast-enhanced MRI images of hepatic IPTs in adults have exhibited similarities to hepato-cholangiocarcinomas (10, 20, 21). However, misclassifying hepatic IPTs as malignant tumors may lead to unnecessary surgical interventions (22), underscoring the critical importance of accurate diagnosis.

The definitive diagnosis of IPT relies on pathological examination. Hepatic IPTs can be pathologically categorized into fibrohistiocytic and lymphoplasmacytic types. The lymphoplasmacytic type, characterized by diffuse lymphoplasmacytic infiltration with numerous IgG4-positive plasma cells, is associated with IgG4-RD (1).The 2020 revised comprehensive diagnostic criteria for IgG4-RD consists of three major items: 1) one or more organs show diffuse or localized swelling or a mass or nodule characteristic of IgG4-RD; 2) elevated serum IgG4 concentrations greater than 135 mg/dl; 3) dense lymphocyte and plasma cell infiltration, defined as > 10 IgG4+ cells per HPF and a > 40% ration of IgG4+/IgG+ cells, accompanied by fibrosis and typical tissue fibrosis, particularly storiform fibrosis, or obliterative phlebitis (23, 24). In our case, a hepatic mass was found, and the serum IgG4 concentration in our case was elevated at 154mg/dl, surpassing the threshold of > 135 mg/dl. Additionally, the pathological features of our cases were consistent with IgG4-RD. The amalgamation of hepatic mass, serum IgG4 concentration, and pathological features led to the diagnosis of IgG4-related hepatic IPT. Besides, the patient presented swelling of multiple superfical lymph nodes in our case. Due to the presence of fungal pneumonia in the patient, multiple superficial lymphadenopathy may be associated with infection. However, since we did not perform pathological biopsies on these lymph nodes, we cannot completely rule out IgG4 related lymph node diseases.

The differential diagnosis of IPT encompasses various conditions, including infection, inflammatory myofibroblastic tumor (IMT), IPT-like follicular dendritic cell (FDC) tumor, inflammatory angiomyolipoma, lymphoma, and inflammatory hepatocellular carcinoma (25). Infections typically manifest with clinical signs such as fever, elevated neutrophil counts, multifocality, and responsiveness to antibiotics. IMT, often initially misdiagnosed as IPT due to similar constitutional symptoms and gross features, accounts for less than 10% of IMTs, and the number of IgG4-positive plasma cells and the IgG4:IgG ratio in IMT are lower than the threshold for IgG-RD. Additionally, the inflammatory infiltrate in IMT is less prominent in peritumoral tissue (26–28). Historically, EBV-positive FDC tumor was considered a subtype of IPT in the 1990s due to similar histological characteristics (29). However, EBV expression is now recognized as a sensitive screening method for distinguishing IPT from FDC tumor. In cases of acute myelocytic leukemia(AML) with spindled myocytes, the heavy but variable infiltrate of lymphocytes, plasma cells, and histiocytes can mimic IPT. However, AML is rarely found in the liver, and hepatic AML rarely exhibits exuberant inflammation (30). Hodgkin lymphoma can sometimes mimic IPT, but lymphoma patients rarely present with hepatic involvement. The presence of scattered large atypical lymphoid cells and markers for B-cells are apparent differences between lymphoma and IPT (31). In the case of inflammatory hepatocellular carcinoma, a biopsy from a region with a paucity of neoplastic cells and massive infiltration of immune cells may resemble an IPT. Immunohistochemical results can help distinguish inflammatory hepatocellular carcinoma from hepatic IPT (25). Considering the clinical signs, the number of IgG4-positive plasma cells, the IgG4:IgG ratio, negative EBV expression, and the inflammatory infiltrate in our case, we successfully ruled out infections and neoplasms that mimic hepatic IPT.

In the management of IgG4-RD, corticosteroids stand as the primary therapeutic approach, showcasing dramatic clinical responses in the majority of cases (32). The recommended corticosteroid regimen involves prednisolone or prednisone at a dose of 0.4-0.6 mg/kg/d for 3-4 weeks, gradually tapering down for maintenance treatment (33). However, a notable tendency to relapse during the maintenance phase has been observed (34, 35). Studies suggest that low-dose corticosteroids administered for 1-3 years may reduce the relapse rate (32). Nevertheless, recent reports on adverse events associated with corticosteroids have constrained their long-term use in IgG4-RD (36). Consequently, the combination of corticosteroids with immunosuppressive agents (IM) has been contemplated for its potential to yield higher remission rates and lower relapse rates compared to corticosteroid monotherapy (37). Mycophenolate mofetil and azathioprine are the most widely used IM in clinical practice (38).A randomized clinical trial showed efficacy and safety of glucocorticoid combined with mycophenolate mofetil therapy for IgG4-RD (39). For this reason, the treatment regimen for the patient in this case involved prednisone in combination with mycophenolate mofetil. Remarkably, the patient exhibited resolution of fever, reduction in the size of the hepatic mass, improvement in markers of inflammation (i.e., ESR and CRP level), and a decline in serum IgG4 levels (665.5 mg/L, normal range: 10-537 mg/L) over an 8-month follow-up period.

Concerning the prognosis, studies indicate that the recurrence rate of IgG4-RD during the follow-up period ranges from 24% to 63%, with variations noted in different investigations (34, 35, 40, 41). A recent study employing Cox regression analysis identified several factors significantly associated with relapse, including higher baseline serum IgG4 concentrations (> 27000 mg/L), baseline elevation of serum eosinophils, multi-organ involvement (more than four organs), lymph node involvement, and higher RI scores (24). Notably, a history of recurrence and severe disease manifestations on imaging are indicative of a higher likelihood of relapse (42). Considering the results of the patient in our report, the favorable outcomes observed, including resolution of symptoms, reduction in the size of the hepatic mass, and improvements in inflammatory markers and serum IgG4 levels, suggest a potentially favorable nonrelapsed prognosis.

## 4 Conclusion

In this report, we present a case of IgG4-related hepatic IPT. The challenges in diagnosis underscore the necessity and complexity of differential diagnosis in patients with a hepatic mass. Histopathological examination remains pivotal for confirming IgG4-related hepatic IPT. In our case, anemia emerged as the primary and non-atypical clinical symptom. Despite the rarity of IgG4-related hepatic IPT, particularly in children, comprehensive testing is imperative to either rule out or confirm the diagnosis when confronted with an unidentified hepatic mass.

## Data availability statement

The original contributions presented in the study are included in the article/**Supplementary Material**. Further inquiries can be directed to the corresponding author.

## Ethics statement

The studies involving humans were approved by Ethics Committee of Jiangxi Provincial Children’s Hospital. The studies were conducted in accordance with the local legislation and institutional requirements. Written informed consent for participation in this study was provided by the participants’ legal guardians/next of kin. Written informed consent was obtained from the individual(s), and minor(s)’ legal guardian/next of kin, for the publication of any potentially identifiable images or data included in this article.

## Author contributions

QW: Writing – original draft, Writing – review & editing. ZX: Writing – original draft, Writing – review & editing. XL: Writing – review & editing. ZW: Writing – review & editing. QZ: Writing – review & editing. CW: Writing – original draft, Writing – review & editing.

## Supplementary material

Supplementary Figure 1 Immunohistochemical analysis of the focal hepatic lesion. All inflammatory cells in the lesion stained diffusely positive for CD38 (**A**, CD38 stain, magnification power: ×200) and Mum-1 (**B**, Mum-1 stain, magnification power: ×200), indicative of plasma cells. Plasma cells were positive for IgG (**C**, IgG stain, magnification power: ×200), with a few plasma cells in the lesion showing focal positivity for IgG4 (>10 plasma cells/high-power field) (**D**, IgG4 stain, magnification power: ×200). The ratio of IgG4/IgG plasma cells was >40%.
