# Supplementary figures and images for: A case report of IgG4-related hepatic inflammatory pseudotumor in a 3-year old boy
Source: Front Immunol. 2024 Apr 30;15:1376276. doi: 10.3389/fimmu.2024.1376276 (PMC11091244; doi:10.3389/fimmu.2024.1376276)

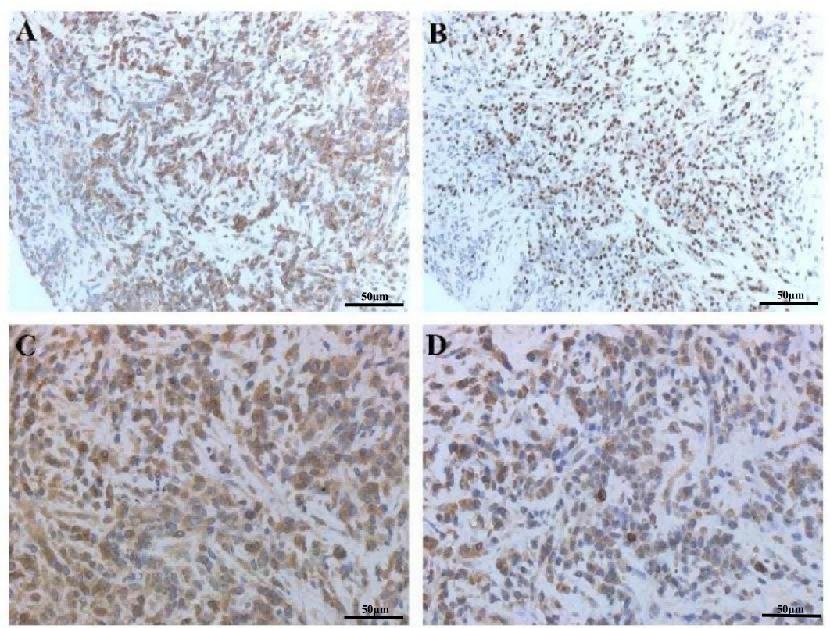

Supplement: Supplementary Figure 1 — Immunohistochemical analysis of the focal hepatic lesion. All inflammatory cells in the lesion stained diffusely positive for CD38 (A, CD38 stain, magnification power: ×200) and Mum-1 (B, Mum-1 stain, magnification power: ×200), indicative of plasma cells. Plasma cells were positive for IgG (C, IgG stain, magnification power: ×200), with a few plasma cells in the lesion showing focal positivity for IgG4 (>10 plasma cells/high-power field) (D, IgG4 stain, magnification power: ×200). The ratio of IgG4/IgG plasma cells was >40%. [file Image_1.jpeg]
